# Correction: *Dlk1-Dio3* locus-derived LncRNAs perpetuate postmitotic motor neuron cell fate and subtype identity

**DOI:** 10.7554/eLife.55647

**Published:** 2020-02-05

**Authors:** Ya-Ping Yen, Wen-Fu Hsieh, Ya-Yin Tsai, Ya-Lin Lu, Ee Shan Liau, Ho-Chiang Hsu, Yen-Chung Chen, Ting-Chun Liu, Mien Chang, Joye Li, Shau-Ping Lin, Jui-Hung Hung, Jun-An Chen

Yen Y-P, Hsieh W-F, Tsai Y-Y, Lu Y-L, Liau ES, Hsu H-C, Chen Y-C, Liu T-C, Chang M, Li J, Lin S-P, Hung J-H, Chen J-A. 2018. Dlk1-Dio3 locus-derived lncRNAs perpetuate postmitotic motor neuron cell fate and subtype identity. *eLife* **7**:e38080. doi: 10.7554/eLife.38080.

Published 12, October 2018

We recently discovered an error in the original Figure 3A, in which the RIP quantification figure in Suz12 was accidentally duplicated from Ezh2. This occurred inadvertently during the conversion of histogram plot to dot format with mislabeled file name of raw data. The figure has been corrected so that the corrected Figure 3A now reflects the correct quantification. The correction does not affect or change any of the conclusions of the manuscript. We apologize for the mistake and any inconvenience or confusion this error may have caused:

Corrected Figure 3

**Figure 1:**
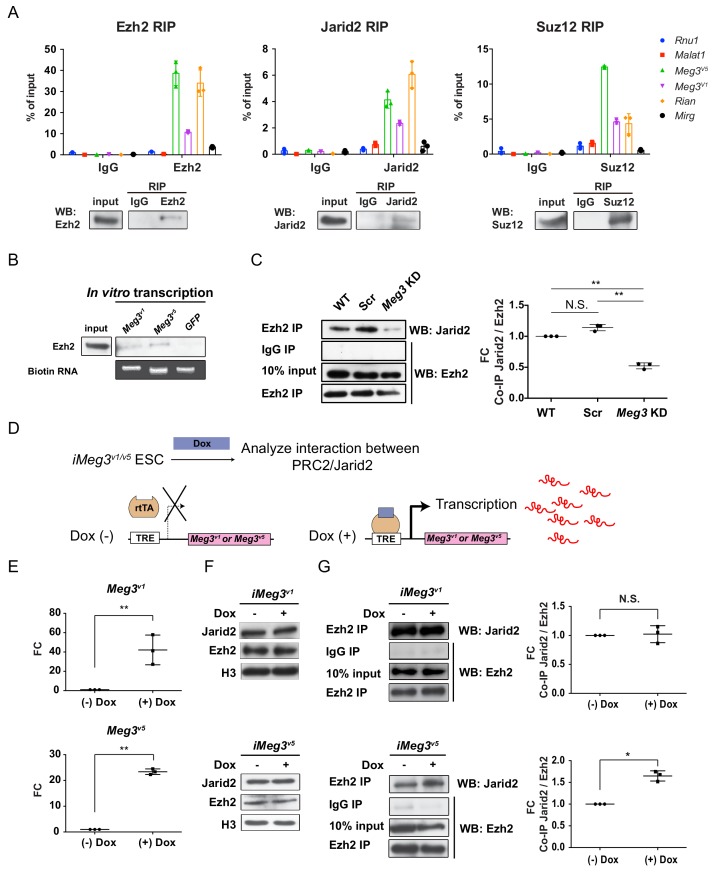


Original Figure 3

**Figure 2:**
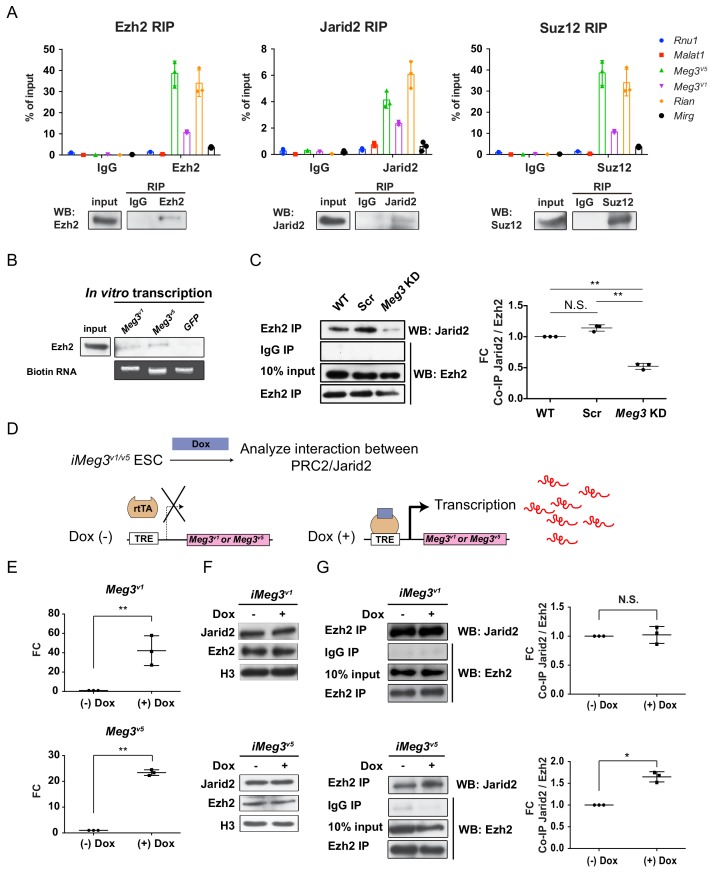


The article has been corrected accordingly.

